# Epacadostat stabilizes the apo-form of IDO1 and signals a pro-tumorigenic pathway in human ovarian cancer cells

**DOI:** 10.3389/fimmu.2024.1346686

**Published:** 2024-01-25

**Authors:** Sofia Rossini, Sara Ambrosino, Claudia Volpi, Maria Laura Belladonna, Maria Teresa Pallotta, Eleonora Panfili, Chiara Suvieri, Antonio Macchiarulo, Giada Mondanelli, Ciriana Orabona

**Affiliations:** ^1^ Department of Medicine and Surgery, University of Perugia, Perugia, Italy; ^2^ Department of Pharmaceutical Sciences, University of Perugia, Perugia, Italy

**Keywords:** apo-IDO1, non-enzymatic function of IDO1, IDO1 plasticity, IDO1 protein degradation, epacadostat, SKOV-3 cells, human ovarian cancer cells, moonlighting protein

## Abstract

The tryptophan-degrading enzyme indoleamine 2,3-dioxygenase 1 (IDO1) is a plastic immune checkpoint molecule that potently orchestrates immune responses within the tumor microenvironment (TME). As a heme-containing protein, IDO1 catalyzes the conversion of the essential amino acid tryptophan into immunoactive metabolites, called kynurenines. By depleting tryptophan and enriching the TME with kynurenines, IDO1 catalytic activity shapes an immunosuppressive TME. Accordingly, the inducible or constitutive IDO1 expression in cancer correlates with a negative prognosis for patients, representing one of the critical tumor-escape mechanisms. However, clinically trialed IDO1 catalytic inhibitors disappointed the expected anti-tumor efficacy. Interestingly, the non-enzymatic apo-form of IDO1 is still active as a transducing protein, capable of promoting an immunoregulatory phenotype in dendritic cells (DCs) as well as a pro-tumorigenic behavior in murine melanoma. Moreover, the IDO1 catalytic inhibitor epacadostat can induce a tolerogenic phenotype in plasmacytoid DCs, overcoming the catalytic inhibition of IDO1. Based on this recent evidence, IDO1 plasticity was investigated in the human ovarian cancer cell line, SKOV-3, that constitutively expresses IDO1 in a dynamic balance between the holo- and apo-protein, and thus potentially endowed with a dual function (i.e., enzymatic and non-enzymatic). Besides inhibiting the catalytic activity, epacadostat persistently stabilizes the apo-form of IDO1 protein, favoring its tyrosine-phosphorylation and promoting its association with the phosphatase SHP-2. In SKOV-3 cells, both these early molecular events activate a signaling pathway transduced by IDO1 apo-protein, which is independent of its catalytic activity and contributes to the tumorigenic phenotype of SKOV-3 cells. Overall, our findings unveiled a new mechanism of action of epacadostat on IDO1 target, repositioning the catalytic inhibitor as a stabilizer of the apo-form of IDO1, still capable of transducing a pro-tumorigenic pathway in SKOV-3 tumor. This mechanism could contribute to clarify the lack of effectiveness of epacadostat in clinical trials and shed light on innovative immunotherapeutic strategies to tackle IDO1 target.

## Introduction

In the last decades, anticancer immunotherapy has provided a variety of approaches for arresting tumor growth (1). Immune checkpoint inhibitors (ICIs) represent one of these therapeutic options that have profoundly improved the clinical management of cancer patients. Unfortunately, a large proportion of patients do not respond or quickly become resistant to approved ICIs and other clinically trialed ICIs have failed to demonstrate efficacy, calling out the urgent need to better elucidate the plasticity of the tumor microenvironment (TME).

Indoleamine 2,3-dioxygenase 1 (IDO1) is an intracellular immune checkpoint highly expressed by both tumor and immune cells in the TME, where it mediates the ‘off’ signal that blocks T cells from killing cancer cells. IDO1 is a heme-containing dioxygenase that controls the metabolic conversion of tryptophan into a series of endogenous metabolites, collectively known as kynurenines (2). IDO1’s catalytic action causes significant immunosuppressive effects in TME because of the reduction of the essential amino acid tryptophan and the accumulation of immune-active kynurenines, which promote the differentiation of T regulatory cells (Tregs), the suppression of effector T and NK cells, and the differentiation of tolerogenic dendritic cells (DCs), with a resulting tumor immune tolerance (3, 4). Accordingly, the pharmacological inhibition of IDO1 has been proposed as an anticancer strategy for removing the ‘off’ signal mediated by IDO1 in the TME. To date, most IDO1 catalytic inhibitors have failed during the clinical trials and none have been approved so far, despite a strong rationale for IDO1 inhibition. Over the last 15 years, more than 50 crystal structures of IDO1 have been published, and a variety of catalytic inhibitors have been developed, yielding a wealth of information on the structure and biology of IDO1 target (5).

As a heme-containing protein, IDO1 is equipped with a high plasticity documented in different cell types (6). The shift between the holo-conformation (namely, the protein containing the heme cofactor) and the apo-conformation (namely, the protein without the heme cofactor) confers to IDO1 protein a flexible immunoregulatory ‘power’ in the TME, depending on the environment requirements. While holo-IDO1 has a metabolic function related to its catalytic activity, apo-IDO1 has a transducer activity mediated by its interaction with SH2-containing proteins, as the Src homology 2 domain phosphatases (SHPs), the phosphoinositide 3-kinase (PI3K), and the suppressor of cytokine signaling 3 (SOCS3) (7–9). Like an ordinary transducing molecule, IDO1 contains two immunoreceptor tyrosine-based inhibitory motifs (ITIMs) in the small domain of the protein that can be tyrosine-phosphorylated by the Src kinase and become docking sites for the interaction with downstream SH2-containing proteins (10). The first evidence supporting a potential non-enzymatic activity has been recently described also for IDO2, a paralogue of IDO1 protein, in the human lung adenocarcinoma cell line A549, suggesting a shared ‘moonlighting’ feature by tryptophan-degrading enzymes (11). The catalytic and signaling functions of IDO1 appear to be confined to mutually exclusive conformations of the protein, compatible with the holo- and apo-IDO1 conformations (12). Overall, apo-IDO1 is no longer just a transient conformation for heme cofactor acquisition that renders the protein enzymatically active, but represents a signaling molecule that could become an attractive target, especially in the tumor cell.

The signaling activity of IDO1 was first observed in DCs where it is responsible for a long-term tolerogenic phenotype that inhibits the Ag-specific immune response (7). An active role of apo-IDO1 in tumor progression was described for the first time in a preclinical model of glioblastoma (13), as well as of melanoma (14), where the non-enzymatic mutant of IDO1 respectively suppresses the anti-tumor immunity and triggers the intrinsic cancer cell proliferation, both independently of the catalytic activity (14).

Among the growing number of IDO1 catalytic inhibitors that exploit the structural flexibility of the IDO1 target (5), the potent and selective inhibitor epacadostat has reached the most advanced phase III clinical stage in metastatic melanoma patients to study the synergistic efficacy in combination with pembrolizumab. Unfortunately, epacadostat did not demonstrate any advantageous benefit compared to pembrolizumab monotherapy (15, 16). Of note, the non-catalytic activity of IDO1 has been postulated as one of the potential reasons for the clinical failure of epacadostat in the Phase III ECHO-301 trial (17). Interestingly, Panfili et al. have recently shown that epacadostat promotes the interaction of IDO1 with its molecular partners SHP phosphatases and PI3K, thereby inducing a tolerogenic phenotype in plasmacytoid DCs that inhibits the Ag-specific response, overcoming the catalytic inhibition of IDO1 (18).

Based on the structural plasticity of IDO1 protein that easily shifts the balance between holo- and apo-IDO1, and the recent findings suggesting a role of the non-enzymatic function of IDO1 in the tumor progression (14), in the current study we focused on the capacity of epacadostat to affect IDO1 conformations ― and thus, its enzymatic/non-enzymatic functions ― in the human ovarian cancer cell line SKOV-3, endogenously expressing human IDO1 in both conformations (19). As the holo- and the apo-IDO1 mediate two different immunoregulatory mechanisms, we wondered whether epacadostat can forge the tumorigenic phenotype of SKOV-3 cells with a mechanism independent of the catalytic inhibition of IDO1.

## Materials and methods

### Cell line, cell treatments, and reagents

The human ovarian adenocarcinoma cell line SKOV-3, purchased from CLS Cell Lines Service GmbH, was grown in RPMI-1640 medium supplemented with 10% FCS and maintained in a humidified atmosphere at 37°C in 5% CO_2_. Cells were routinely screened to confirm the absence of mycoplasma contamination. For cellular assays, SKOV-3 cells were seeded at the final concentration of 0.15 x 10^6^ cells/ml in 12-well plate, unless otherwise specified, and then exposed to epacadostat for the indicated times. Epacadostat was purchased from SelleckChem and was used at the final concentration of 1 µM unless otherwise indicated. Cycloheximide was obtained from Sigma-Aldrich and used at the final concentration of 50 µg/ml. For cycloheximide-chase assay, cells were pre-treated with the protein synthesis inhibitor for 1 hour prior the addition of epacadostat or vehicle. Cells were then incubated up to 8 and 16 hours for the short and long kinetics analysis, respectively. For washout experiments, SKOV-3 cells were pre-conditioned with epacadostat or vehicle for 16 hours at 37°C, and then washed twice with medium to completely remove the stimulus. Then, cells were exposed to cycloheximide and incubated for 4 or 8 hours (post-washout) to measure IDO1 protein expression and kynurenine concentration. DMSO, used as control, was purchased from Sigma-Aldrich. IDO1 knockdown experiments were performed by using Silencer^®^ Select human *IDO1* siRNA (ID s7425) and Silencer^®^ Select Negative Control No.1 siRNA from Thermo Fischer Scientific, according to the manufacturer’s instructions. Briefly, SKOV-3 cells were incubated overnight at 37°C at the final concentration of 0.1 x 10^6^ cells/ml in 6-well plate. The day after, cells were transfected with 75 pmol of *IDO1*-specific or negative control siRNAs, by using Lipofectamine™ 3000 Transfection Reagent (Thermo Fischer Scientific). After 48 hours, IDO1-silenced SKOV-3 cells were used for the scratch wound healing assay and assessed for IDO1 silencing, by analyzing IDO1 protein expression and catalytic activity, as represented in the experimental workflow in **Supplementary Figure 2A**.

### Immunoprecipitation experiments

IDO1 tyrosine-phosphorylation and its interaction with SHP-2 were assessed by immunoprecipitation experiments with 2.5 x 10^6^ cells/sample. Cells were lysed in M-PER buffer (Thermo Fisher Scientific), containing protease and phosphatase inhibitor cocktails (Thermo Fischer Scientific), and a representative aliquot per sample was collected as a whole cell lysate (WCL) control. The remaining lysates were used for immunoprecipitation studies, performed following the manufacturer’s protocol (Thermo Fisher Scientific) and as previously described (14). For each sample, 12.5 μl of magnetic beads (Dynabeads Protein G, Thermo Fischer Scientific) were blocked with PBS containing 0.5% BSA (w/v) and conjugated overnight either with an anti-phospho-tyrosine (p-Tyr-1000) MultiMab™ Rabbit monoclonal antibodies mix (Cell Signaling Technology), or a mouse monoclonal anti-SH-PTP2 antibody (clone B-1, Santa Cruz Biotechnology), for IDO1 tyrosine-phosphorylation and SHP-2/IDO1 interaction analyses, respectively. Beads without the specific antibody were used for negative control samples. Antibody-conjugated beads were then incubated with cell lysates, overnight at 4°C, and then washed with washing buffer (25 mM citric acid, 50 mM dibasic sodium phosphate dodecahydrate, pH 5). Subsequently, the immuno-complexes were eluted with elution buffer (0.1 M sodium citrate dihydrate, pH 2-3) plus Laemmli buffer containing 2-mercaptoethanol and used for western blotting analysis.

### Immunoblot and kynurenine measurement

IDO1 protein expression was analyzed by means of a rabbit monoclonal anti-human IDO1 antibody (D5J4E™, Cell Signaling Technology), whereas SHP-2 protein level was detected by the use of a mouse monoclonal anti-SH-PTP2 antibody (clone B-1, Santa Cruz Biotechnology). Moreover, a mouse monoclonal anti-β-tubulin antibody (clone AA2, Sigma-Aldrich) was used as normalizer. SKOV-3 cells were lysed in Laemmli buffer containing 2-mercaptoethanol and used for sodium dodecyl sulfate/polyacrylamide gel electrophoresis (SDS/PAGE). After incubation with the specific horseradish peroxidase-conjugated secondary antibodies (Sigma-Aldrich) and the addition of Clarity™ Western ECL (Enhanced ChemiLuminescence, Bio-Rad Laboratories) substrate, signals were detected by the use of ChemiDoc™ MP Imaging System (Bio-Rad Laboratories). Protein expression was quantified by densitometric analysis, using ImageLab Software (Bio-Rad Laboratories), as previously described (14). The enzymatic activity of IDO1 was evaluated in cell culture supernatants in terms of the ability of the enzyme to metabolize tryptophan into kynurenine, by HPLC analysis as previously described (20).

### Real-Time PCR analysis

For gene expression analysis, SKOV-3 cells were treated with epacadostat for 24 hours and lysed in TRIzol reagent (Thermo Fischer Scientific) to isolate RNA, according to the manufacturer’s instructions. One µg of RNA was reverse-transcribed using QuantiTec Reverse Transcription Kit (Qiagen) and analyzed by Real-Time PCR, using Stratagene Mx3005P (Agilent Technologies). Specific primers were used to determine the expression of the human *IDO1* gene (sense, 5’-TCACAGACCACAAGTCACAG-3’; antisense, 5’-GCAAGACCTTACGGACATCT-3’). Data were calculated as the ratio of the gene of interest to that of human β*-*actin expression (*ACTB*) (sense, 5’-CTCGTCGTCGACAACGGCT-3’; antisense, 5’-TCAGGGTGAGGATGCCTCTC -3’) by the relative quantification method (ΔΔCt). Values are presented as normalized transcript expression in samples relative to normalized transcript expression in control cells (1-fold).

### Cell viability assay

The cell viability of SKOV-3 cells exposed to epacadostat was analyzed by MTT assay. Cells were seeded at the final concentration of 5 x 10^3^ cells/100 µl/well in a 96-well plate, and treated with epacadostat for 24, 48, and 72 hours. Vehicle-treated cells were used as control. At each time point, the stimulus was removed and cells were provided with 110 µl/well of complete medium containing MTT (Sigma-Aldrich) at the final concentration of 0.45 mg/ml. After 4 hours at 37°C, 100 µl/well of the solubilization buffer (SDS 10% in HCl 0.01 M) were added and cells were incubated overnight at 37°C. The day after, cell viability was determined by measuring the absorbance at 570 nm, by using a UV/visible spectrophotometer (TECAN, Thermo Fisher Scientific).

### Scratch wound healing assay

For the scratch wound healing assay, SKOV-3 cells were seeded at 0.2 x 10^6^ cells/ml in 6-well plate and cultured overnight to reach approximately over 80% of confluence. The day after, a 10-µl sterile pipette tip was used to make a scratch line on the monolayer of confluent cells, at the bottom of the well. Cells were washed twice to remove cellular debris and then treated with epacadostat or DMSO (as control). Cells were incubated at 37°C, in a humidified 5% CO_2_ incubator, for 48 hours. Over this incubation time, the wound healings were continuously observed by using a Nikon Eclipse T*i* inverted microscope (Nikon) with 10X magnification, and pictures were acquired every 4 hours. The area of the wound healing was determined by using the MRI Wound Healing tool (ImageJ Software, NIH) and the data were reported as percentage of the wound closure respect to time 0.

### Soft agar colony formation assay

Anchorage-independent growth of SKOV-3 cells was studied by performing a soft agar colony formation assay, after conditioning cells with epacadostat for 16 hours. Firstly, the bottom of a 6-well plate was precoated with a mix (1:1 ratio) of 1% agarose solution and 2X complete RPMI-1640 medium (1.5 ml/well). Then, pre-conditioned cells were resuspended in 2X complete RPMI-1640 medium (containing epacadostat or vehicle) and 0.6% agarose solution (1:1 ratio) and plated (5 x 10^3^ cells/well) on the precoated 6-well plate (1.5 ml/well). The stimulus renewal was performed every other day, by the addition of 200 µl of complete medium (containing epacadostat or DMSO) over the agarose’s upper layer. After 21 days, colonies were imaged by using a Nikon Eclipse T*i* inverted microscope (Nikon) with 40X magnification. The size of colonies was analyzed in 30 randomly selected visual fields in each well and measured using the NIS-Elements AR Software (Nikon). Subsequently, the colonies were stained by adding MTT, at the final concentration of 1 mg/ml, and incubated overnight at 37°C.

### Statistical analysis

All analyses were performed using Prism version 10.0.3 (GraphPad Software). Data were shown as mean ± SD of at least three independent experiments. A P value less than 0.05 was considered significant. Data were analyzed by 2-tailed unpaired Student’s *t*-test or two-way ANOVA followed by *post-hoc* Bonferroni’s test when three or more samples were under comparison. The IC_50_ and EC_50_ were calculated by nonlinear regression analysis of raw data from the concentration-response curve, by applying the “log[inhibitor] *vs* response” and “log[agonist] *vs* response” equations, respectively. The negative logarithm of the IC_50_ and EC_50_ in unit of Molar was used to express the pIC_50_ and pEC_50_ values. The half-life (t_1/2_) and the degradation speed (K) of IDO1 protein were calculated by nonlinear regression analysis of raw data, by using the “one-phase decay” equation.

## Results

### Epacadostat increases IDO1 protein expression in SKOV-3 cells

**Figure 1:**
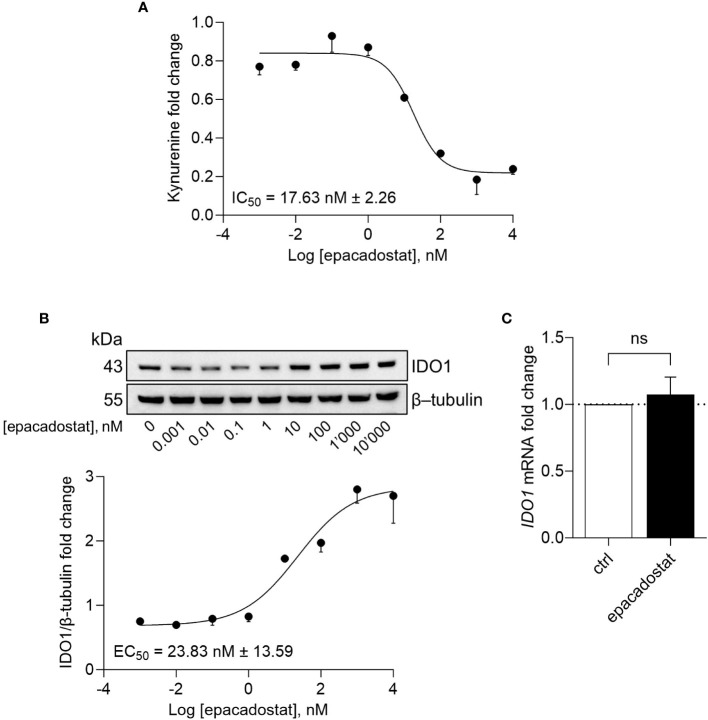
Epacadostat inhibits IDO1 catalytic activity and increases IDO1 protein expression. **(A)** Kynurenine released by SKOV-3 cells treated with increasing concentration of epacadostat (from 10^-3^ to 10^4^ nM), for 24 hours. Results are shown as kynurenine fold change of epacadostat-treated *versus* vehicle-treated samples (1-fold). Epacadostat IC_50_ = 17.63 nM ± 2.26 (pIC_50_ = 7.76 ± 0.06). **(B)** Immunoblotting analysis of IDO1 protein expression in lysates from SKOV-3 cells treated as in **(A)**. β-tubulin expression was used as normalizer. One representative immunoblot of three is shown. The IDO1/β-tubulin protein ratio of scanning densitometry analysis is reported as fold change of epacadostat-treated *versus* vehicle-treated cells (1-fold). Epacadostat EC_50_ = 23.83 nM ± 13.59 (pEC_50_ = 7.66 ± 0.26). **(C)** Real-Time PCR analysis of *IDO1* transcript in SKOV-3 cells treated with epacadostat (1 µM) for 24 hours, normalized to the expression of *ACTB* and reported as relative to results in vehicle-treated cells (ctrl; dotted line, 1-fold). Data in **(A-C)** are mean ± SD of three independent experiments. Data in **(C)** were analyzed by unpaired Student’s *t*-test. ns, not significant.

Besides being unable to degrade tryptophan, the apo-IDO1 assumes a conformation prone for transducing an intracellular signal, as previously demonstrated in tumor cells ectopically expressing a loss-of-function mutant of IDO1 (namely, the apo-form) as well as in response to the IDO1 catalytic inhibitor epacadostat (13, 14, 18). To investigate the effect of epacadostat on endogenous IDO1, we resorted to the human ovarian cancer cell line SKOV-3 that constitutively expresses the highest level of *IDO1* transcript among different human ovarian cancers (**Supplementary Figure 1A**) (21). Moreover, in SKOV-3 cells, IDO1 protein is present in a dynamic balance between the apo- and the holo-form (19). By treating cells with increasing concentration of the molecule, we confirmed that epacadostat inhibited the catalytic activity of IDO1 with a pIC_50_ of 7.76 ± 0.06 (**Figure 1A**). Of note, we found that the protein level of IDO1 increased in the cells exposed to epacadostat with a pEC_50_ of 7.66 ± 0.26 (**Figure 1B**), in the same concentrations range as pIC_50_ for the catalytic inhibition, suggesting a dual effect of epacadostat on IDO1. Interestingly, the catalytic inhibitor linrodostat also exerts a similar dual effect on IDO1 protein (data not shown). Thus, to investigate whether epacadostat affected the transcript level of *IDO1*, SKOV-3 cells were treated with a fixed concentration of the molecule and the *IDO1* mRNA level was evaluated. Based on the previous literature (18, 22), we selected a final concentration of 1 µM, that has proved to be not cytotoxic (**Supplementary Figure 1B**) and able to inhibit IDO1 catalytic activity (**Supplementary Figure 1C**) as well as increase IDO1 protein expression in SKOV-3 (**Supplementary Figure 1D**). Results from Real-Time PCR analysis demonstrated that the level of *IDO1* was not significantly different in cells exposed to epacadostat and the control counterpart (**Figure 1C**), suggesting that post-translational regulatory mechanisms underlie the sustained IDO1 protein levels observed in SKOV-3 in response to epacadostat.

### Epacadostat stabilizes the apo-form of IDO1 protein in SKOV-3 cells

**Figure 2:**
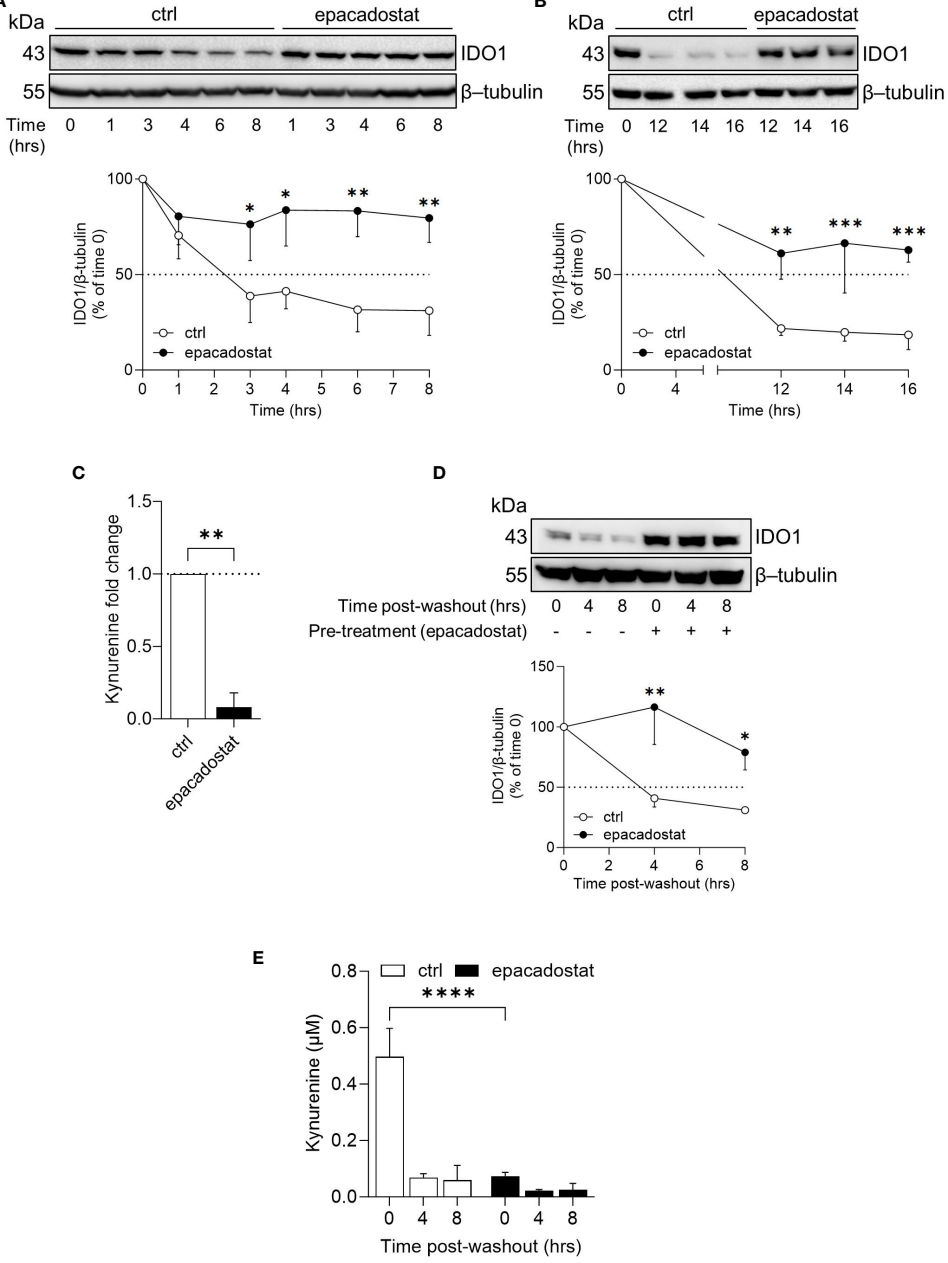
Epacadostat increases the half-life of IDO1 protein in SKOV-3 cells. **(A)** Cycloheximide-chase assay followed by immunoblotting analysis of IDO1 protein expression in lysates from SKOV-3 cells pre-treated with cycloheximide (50 μg/ml) for 1 hour and then exposed to epacadostat (1 µM) for the indicated time (from 0 to 8 hours). For each time point, vehicle-treated cells were used as control (ctrl). β-tubulin expression was used as normalizer. The exponential decay regression analysis of the IDO1/β-tubulin protein ratio is expressed as percentage of time 0 (time 0 = 100%; dotted line, 50%). **(B)** Immunoblotting analysis of SKOV-3 cells treated as in **(A)** for a longer time course (from 0 to 16 hours). β-tubulin expression was used as normalizer. Data were expressed as described in **(A)**. **(C)** Kynurenine released by SKOV-3 cells treated as in **(B)** for 16 hours. Results are shown as kynurenine fold change of cycloheximide/epacadostat-treated *versus* cycloheximide/vehicle-treated samples (dotted line, 1-fold). **(D)** Immunoblotting analysis of IDO1 protein expression in lysates from SKOV-3 cells pre-conditioned with epacadostat (1 µM) or vehicle (ctrl) for 16 hours. Cells were then washed and exposed to cycloheximide (50 μg/ml) for the indicated times (post-washout). β-tubulin expression was used as normalizer. The IDO1/β-tubulin protein ratio is expressed as percentage of the respective time 0 (time 0 = 100%; dotted line). **(E)** Kynurenine levels (µM) in cell culture supernatants from SKOV-3 cells treated as in **(D)**. Data in **(A–E)** are mean ± SD of three independent experiments, and one immunoblot representative of three is shown for **(A, B, D)**. Data in **(A, B, D, E)** were analyzed by two-way ANOVA followed by *post-hoc* Bonferroni’s test; data in **(C)** were analyzed by unpaired Student’s *t*-test. *P < 0.05, **P < 0.01, ***P < 0.001, ****P < 0.0001.

In B16 melanoma cells, the ectopic apo-IDO1 protein has a greater stability compared to the holo-IDO1 protein (14). Thus, we investigated whether epacadostat – by binding IDO1 – favored a protein conformation less prone to post-translational degradation. To this purpose, the kinetic of the intracellular protein turnover was analyzed by a cycloheximide-chase assay followed by immunoblotting. Results indicated that the protein expression of IDO1 remained more stable in SKOV-3 cells treated with epacadostat as compared to control cells (**Figure 2A**) and this effect lasted for at least 16 hours (**Figure 2B**). In particular, the IDO1 half-life is significantly longer in the presence of epacadostat (t_1/2_ > 29.420 hours ± 10.64; degradation speed K, 0.025 hour^−1^ ± 0.01) than that of the vehicle-treated cells (t_1/2_ > 4.787 hours ± 1.63; degradation speed K, 0.154 hour^−1^ ± 0.05) that resulted similar to the IDO1 turnover previously observed in other cell types (23, 24). Moreover, the stabilized IDO1 protein is still catalytically inactive (**Figure 2C**). Interestingly, the complete washout of epacadostat from SKOV-3 culture media – after 16 hours of cell pre-treatment – did not restore the conventional IDO1 protein turnover (**Figure 2D**), nor the catalytic activity of the enzyme (**Figure 2E**). Overall, these findings indicated that the pharmacological effect of epacadostat persists long after the initial exposure and prolongs the half-life of IDO1 protein in the SKOV-3 cells.

### Epacadostat promotes a signaling conformation of IDO1 in SKOV-3 cells

**Figure 3:**
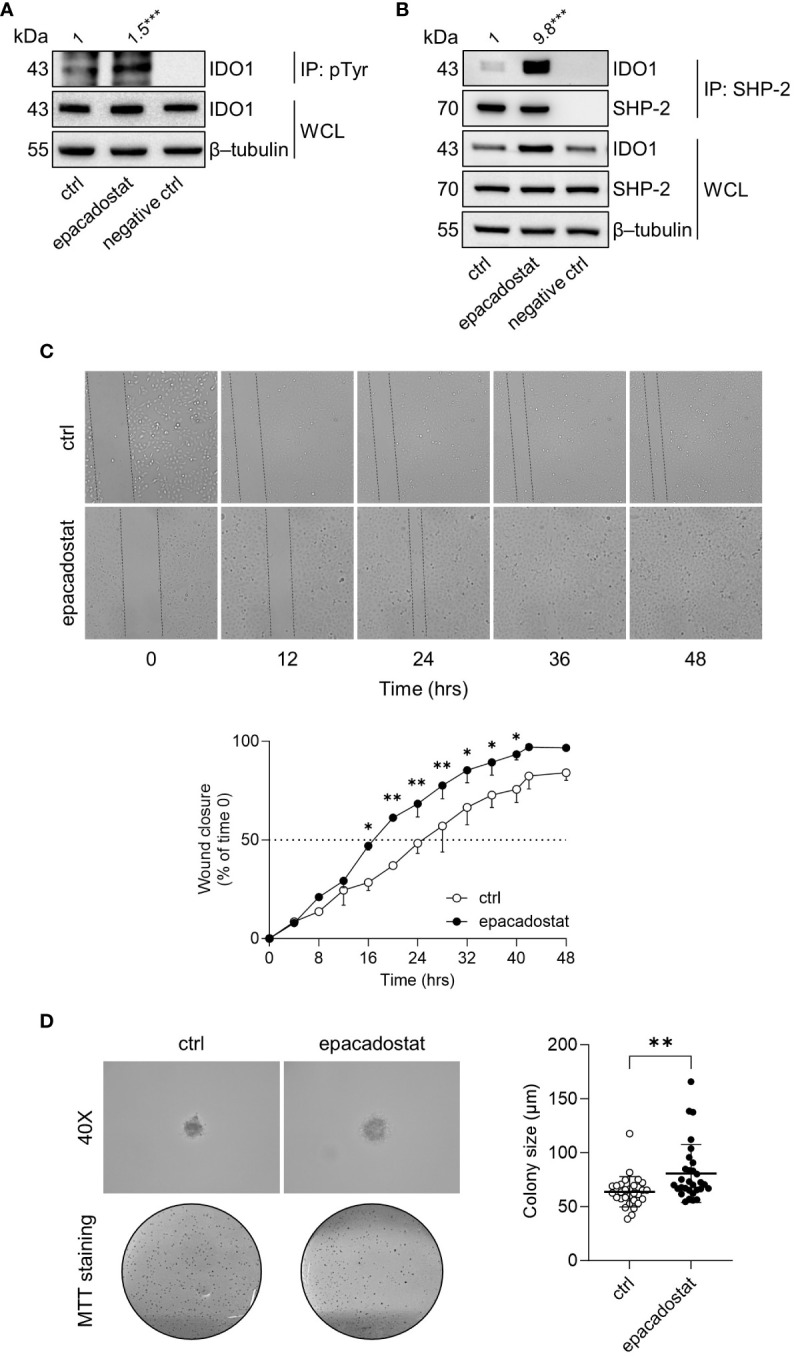
Epacadostat promotes IDO1 phosphorylation and confers a tumorigenic phenotype to SKOV-3 cells. **(A)** p-Tyr immunoprecipitation (IP) and IDO1 immunoblotting analysis performed in lysates from SKOV-3 cells treated with epacadostat (1 μM). Vehicle-treated cells were used as control (ctrl), while immunoprecipitation without antibody was included as negative control. Whole-cell lysates (WCL) was used as control of input IDO1 protein expression, whereas β-tubulin expression was used as normalizer. One representative immunoblot of three is shown. The amount of immunoprecipitated IDO1, measured by densitometric quantification of the specific bands, is reported as fold change of epacadostat-treated *versus* vehicle-treated cells (1-fold), and indicated above the corresponding bands. **(B)** SHP-2 immunoprecipitation (IP) followed by SHP-2 and IDO1 immunoblotting analysis performed in lysates from SKOV-3 cells treated with epacadostat (1 μM) for 16 hours. Vehicle-treated cells were used as control (ctrl), while immunoprecipitation without antibody was included as negative control. WCL was used as control of SHP-2 and IDO1 input protein expression, whereas β-tubulin expression was used as normalizer. One representative immunoblot of three is shown. The amount of co-precipitated IDO1, measured by densitometric quantification, is expressed as IDO1/SHP-2 ratio and reported as fold change of epacadostat-treated *versus* vehicle-treated cells (1-fold), above the corresponding bands. **(C)** Analysis of the wound closure (black dotted lines) on SKOV-3 cells treated with epacadostat (1 µM) or vehicle alone (ctrl), over the time (from 0 to 48 hours). For each reported time point (0, 12, 24, 36, 48 hours), one representative image of three is shown. Data are reported as percentage of the wound closure respect to time 0 (time 0 = 0%; dotted line, 50%). **(D)** Soft agar colony formation assay in SKOV-3 cells pre-treated with epacadostat (1 µM) and then exposed to the stimulus every other day. After 21 days, colonies were imaged at high magnification (40X) and then stained with MTT. Pictures are representative of three independent experiments. Data are reported as colony size (µm) measured in epacadostat-treated and vehicle-treated cells. Data in **(A, B)** are mean of three independent experiments and were analyzed by unpaired Student’s *t*-test. Data in **(C, D)** are mean ± SD of three independent experiments and were analyzed using two-way ANOVA followed by *post-hoc* Bonferroni’s test and unpaired Student’s *t*-test, respectively. *P < 0.05, **P < 0.01, ***P < 0.001.

IDO1 is the prototype of a dynamic protein, as it acquires mutually exclusive conformations endowed with distinct functions, namely the enzymatic and the non-enzymatic forms (12). The phosphorylation of specific tyrosine residues in the ITIMs is the critical event to trigger the IDO1 non-enzymatic function (6, 10). Once phosphorylated, IDO1 interacts with different molecular partners, including the phosphatase SHP-2 (14, 18). Thus, we investigated whether epacadostat stabilized the conformation of IDO1 amenable of phosphorylation and interaction with SHP-2 phosphatase. Results from immunoprecipitation followed by immunoblotting analysis demonstrated that the tyrosine-phosphorylation and the association of IDO1 with SHP-2 significantly increased in SKOV-3 cells in response to epacadostat (1.5 and 9.8 folds, respectively) (**Figure 3A, B**).

SHP-2 is a well-recognized oncogene enabling a broad spectrum of human malignancies, including ovarian cancers (25–27). Thus, we analyzed the effect of epacadostat on SKOV-3 cells phenotype by measuring cellular motility and ability to growth in an anchorage-independent manner. In the wound healing assay, epacadostat accelerated the migratory capacity of SKOV-3 cells by promoting the wound closure faster than the vehicle-treated cells that did not close the wound in 48 hours (**Figure 3C**). Differently, IDO1 knockdown of SKOV-3 cells slowed their migratory capacity compared to IDO1-competent SKOV-3 cells. In fact, IDO1-silenced SKOV-3 cells achieved 50% wound closure after 24 hours compared to negative control siRNA-treated SKOV-3 cells, which only achieved wound closure after 12 hours (**Supplementary Figure 2**), confirming a role of IDO1 protein in the migratory ability of SKOV-3 cells. In addition, epacadostat treatment increased the ability of SKOV-3 cells to form colonies in semisolid media (**Figure 3D**). Overall, these data suggested that epacadostat – although inhibits the catalytic activity of IDO1 – promotes the signal conformation of IDO1 that potentiates *in vitro* the tumorigenic phenotype of SKOV-3 cells.

## Discussion

IDO1 is a cofactor-binding and redox-sensitive protein, characterized by a structural plasticity, which calls for a deeper study of the protein biology in its native cellular environment. Several preclinical mouse tumor models and the worse survival of patients bearing IDO1-expressing tumors support the relevance of IDO1-mediated immune suppression in the TME (28, 29). However, the so far developed strategies of IDO1 inhibition disappointed their postulated anti-tumor efficacy, suggesting the need of developing more efficient ways of blocking IDO1-mediated pathways.

In the last years, a bulk of new evidence on the structural and biological properties of IDO1 has come to the light and entitled IDO1 as a ‘moonlighting’ protein capable of multiple biological functions (30–32). A peculiar feature of moonlighting proteins is the ability to switch from one to the other function by changing the protein conformation according to cell needs. Specifically, besides the well-known enzymatic activity, a non-enzymatic function of IDO1 was described for the first time in murine plasmacytoid DCs, in which it potentiated the immunoregulatory tryptophan metabolism catalyzed by IDO1 (7). A non-enzymatic role of IDO1 was also described in the TME where it promoted tumor progression independently of its catalytic activity (13, 14).

In the human ovarian cancer cell line SKOV-3, IDO1 is endogenously expressed in a dynamical balance between the holo- and the apo-conformation, depending on the intracellular heme availability and factors stabilizing the protein conformation (19, 33). The lability of heme binding in IDO1 plays a significant role in the post-translational control of IDO1 activity in the cells. In the current study, we demonstrated that, in SKOV-3 tumor cells, epacadostat increases and persistently stabilizes IDO1 protein in a catalytically inactive conformation, interestingly maintained even after the epacadostat washout. Although the enzymatic activity is inhibited, the stabilized IDO1 protein is tyrosine-phosphorylated and capable of associating the oncogene SHP-2 phosphatase (25, 26), two early molecular events in the signaling of IDO1 that have been previously demonstrated to incite the progression of B16 melanoma cells (14). Similarly, in SKOV-3 cells treated with epacadostat the signaling triggered by the transducing molecular complex IDO1-SHP-2 accelerates the migratory capacity and the colony-forming ability of SKOV-3 cells, suggesting a pro-tumorigenic phenotype.

These preliminary observations confirm the moonlighting ability of IDO1 to switch from the catalytic to the signaling function, allowing tumor cells to resist the enzymatic inhibition of IDO1 by epacadostat. Both the enzymatic and the non-enzymatic (namely, the signaling) mechanisms of IDO1 are mutually exclusive in SKOV-3 cells, because they require distinct conformations of the protein as previously demonstrated (12). Accordingly, in SKOV-3 cells epacadostat stabilizes a conformation of IDO1 that results catalytically inactive ― as expected by a catalytic inhibitor ―, but capable of transducing a pro-tumorigenic signal in the cell. This pro-tumorigenic effect of epacadostat in the SKOV-3 tumor, together with a recently published effect of epacadostat in murine plasmacytoid DCs, combined with the observation that the non-enzymatic IDO1-mediated functions may play a key role in tumor progression of melanoma and glioblastoma (13, 14), urgently calls for a better understanding of the role of IDO1 in the TME.

Extensive evidence supports the notion that tryptophan catabolism mediated by the IDO1 enzyme is the unique immune regulatory mechanism that promotes tumor growth. However, the development of the catalytic inhibitors of IDO1 has not yet provided a licensed anti-cancer drug and has experienced a significant setback after the failure of epacadostat in phase III trial. Recent evidence (14, 18) and the current study highlight a new prospective on the IDO1 target in cancer immunotherapy, by elucidating the non-enzymatic key role of IDO1 in tumor progression. This could pave the way for the development of a new generation of IDO1 blockers capable of inhibiting both the enzymatic and the transducing functions of IDO1. At this purpose, the proteolysis-targeting chimeras (PROTACs) is an emerging technology to degrade oncogenic proteins via the ubiquitin-proteasome pathway and a viable approach to simultaneously inhibit both IDO1 functions. The first PROTAC degrading IDO1, which conjugates epacadostat to the E3 ligase ligand lenalidomide through a hydrophilic linker, has been reported by Hu et al. (34). Thereafter, an additional IDO1-based PROTAC was reported to be therapeutically active in a mouse model of glioblastoma (35). Alternative IDO1-based therapies are expected to emerge by selectively targeting the non-enzymatic function of IDO1 in tumor cells once the signaling pathway mediated by IDO1 is fully elucidated. Although significant progress has been made in the last decade demonstrating the relevance of a non-enzymatic activity of IDO1 in both immune and tumor cells (7, 14, 18), several issues remain to be addressed, including *i*) to characterize the molecular partners involved in IDO1 signaling in different tumor types in order to optimize synergistic therapies and establish more reliable pharmacodynamic markers; *ii*) to demonstrate a tumor-specificity of IDO1 signaling. In fact, the non-enzymatic function of IDO1 is strictly related to the cell type populating the TME (i.e., tumor, immune, endothelial, and stromal cells). Activation of IDO1 signaling in the tumor cell plays a pro-tumorigenic role rather than immunoregulatory functions. In addition to the IDO1 expression by tumor cells, the picture of the intrinsic pro-tumorigenic IDO1-mediated pathway could contribute to a better selection and stratification of cancer patients for future clinical trials; *iii*) notably, the signaling conformation of IDO1 in tumor cells seems to be more stable than the physiological turnover of the protein, which is reminiscent of an oncogenic property. For instance, the identification of key point mutations on IDO1 critical for protein stability might direct new therapeutic strategies targeting this oncoprotein on a human scale. Therefore, based on the recent observations, the in-depth rewiring of IDO1 in cancer immunology is urgently needed to develop innovative and effective IDO1-targeted therapies against cancer.

## Data availability statement

The original contributions presented in the study are included in the article/**Supplementary Material**. Further inquiries can be directed to the corresponding author.

## Ethics statement

Ethical approval was not required for the studies on humans in accordance with the local legislation and institutional requirements because only commercially available established cell lines were used.

## Author contributions

SR: Data curation, Investigation, Methodology, Writing – original draft. SA: Investigation, Methodology, Writing – original draft. CV: Investigation, Methodology, Validation, Writing – original draft. MB: Investigation, Methodology, Validation, Writing – review & editing. MP: Investigation, Methodology, Validation, Writing – original draft. EP: Investigation, Methodology, Writing – original draft. CS: Investigation, Methodology, Writing – original draft. AM: Conceptualization, Formal Analysis, Supervision, Writing – review & editing. GM: Conceptualization, Supervision, Writing – review & editing. CO: Conceptualization, Funding acquisition, Project administration, Supervision, Writing – review & editing.

## Supplementary material

Supplementary Figure 1 Expression of IDO1 in human ovarian cancers and validation of epacadostat (1 μM) in SKOV-3 cells. **(A)** Expression level of human IDO1 transcript (Ensembl: ENSG00000131203) in different human ovarian cancers, reported as nTPM. Image: Human Protein Atlas. Image/data available from https://www.proteinatlas.org/ENSG00000131203-IDO1/cell+line#ovarian_cancer. **(B)** Cell viability of SKOV-3 cells incubated with epacadostat (1 µM) and monitored for 24, 48, and 72 hours by MTT assay. Vehicle-treated cells were used as control (ctrl). Cell viability is directly proportional to the absorbance measured at 570 nm. **(C)** Kynurenine (µM) released by SKOV-3 cells treated with epacadostat (1 µM) for 24 hours. Vehicle-treated cells were used as control (ctrl). **(D)** Immunoblot analysis of IDO1 protein expression in lysates from SKOV-3 cells treated as in **(C)**. β-tubulin expression was used as normalizer. One representative immunoblot of three is shown. The IDO1/β-tubulin ratio of scanning densitometry analysis is reported as fold change of epacadostat-treated *versus* vehicle-treated cells (ctrl; dotted line, 1-fold). Data in **(B-D)** are mean ± SD of three independent experiments. Data in **(B)** were analyzed by two-way ANOVA followed by *post-hoc* Bonferroni’s test; data in **(C, D)** were analyzed by unpaired Student’s *t*-test. ns, not significant, **P < 0.01, ***P < 0.001.

Supplementary Figure 2 IDO1 knockdown slows down SKOV-3 cells migratory capacity. **(A)** Experimental workflow for IDO1 knockdown, cell validation, and wound healing assay in SKOV-3 cells (Created by BioRender.com). SKOV-3 cells were transfected with *IDO1*-specific or negative control (n.c.) siRNAs (d 0; d, day) and after 48 hours (d +2) were either validated for IDO1 silencing, or used for wound healing assay and monitored for additional 48 hours (d +4). **(B)** Immunoblot analysis of IDO1 protein expression in lysates from SKOV-3 cells after 48 hours of siRNA transfection, as indicated in **(A)**. β-tubulin expression was used as normalizer. One representative immunoblot of two is shown. The IDO1/β-tubulin ratio of scanning densitometry analysis is reported as fold change of *IDO1* siRNA- *versus* n.c. siRNA-transfected cells (dotted line, 1-fold). **(C)** Kynurenine released by SKOV-3 cells treated as in **(B)**. Results are shown as kynurenine fold change of *IDO1* siRNA- *versus* n.c. siRNA-transfected cells (dotted line, 1-fold). **(D)** Analysis of the wound closure (black dotted lines) in SKOV-3 cells treated as indicated in **(A)**, over the time (from 0 to 48 hours). For each reported time point (0, 12, 24, 36, 48 hours), one representative image of three is shown. Data are reported as percentage of the wound closure respect to time 0 (time 0 = 0%; dotted line, 50%). Data in **(B-D)** are mean ± SD of two independent experiments. Data in **(B, C)** were analyzed by unpaired Student’s *t*-test; data in **(D)** were analyzed using two-way ANOVA followed by *post-hoc* Bonferroni’s test. **P < 0.01, ***P < 0.001, ****P < 0.0001.
