# Supplementary figures and images for: Epacadostat stabilizes the apo-form of IDO1 and signals a pro-tumorigenic pathway in human ovarian cancer cells
Source: Front Immunol. 2024 Jan 25;15:1346686. doi: 10.3389/fimmu.2024.1346686 (PMC10850306; doi:10.3389/fimmu.2024.1346686)

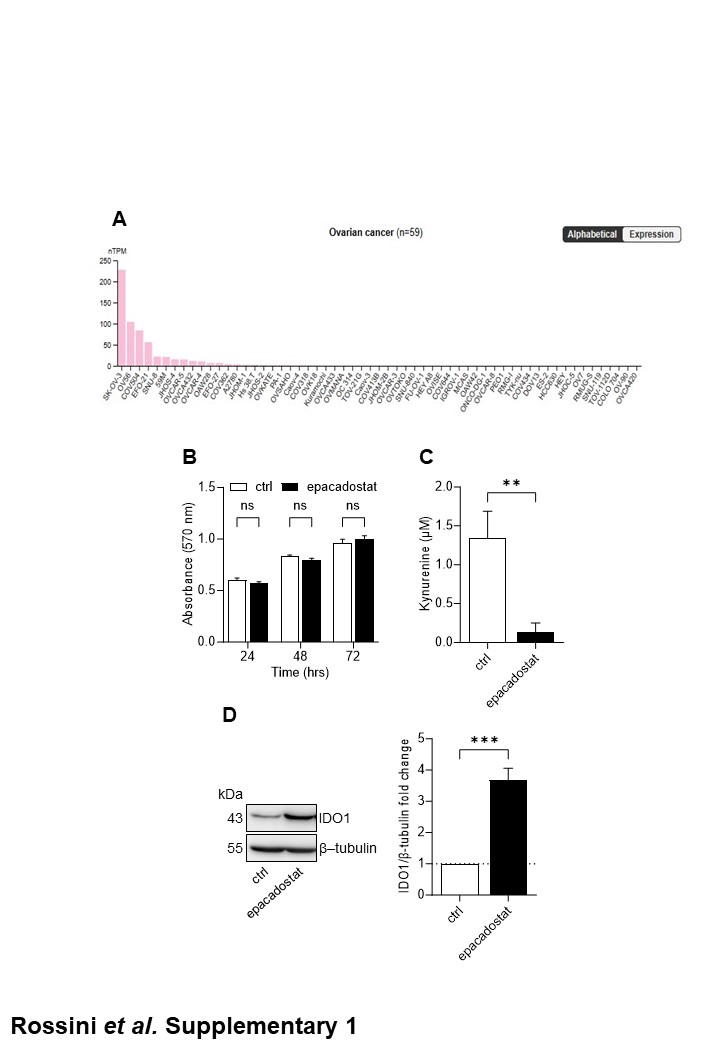

Supplement: Supplementary Figure 1 — Expression of IDO1 in human ovarian cancers and validation of epacadostat (1 μM) in SKOV-3 cells. (A) Expression level of human IDO1 transcript (Ensembl: ENSG00000131203) in different human ovarian cancers, reported as nTPM. Image: Human Protein Atlas. Image/data available from https://www.proteinatlas.org/ENSG00000131203-IDO1/cell+line#ovarian_cancer. (B) Cell viability of SKOV-3 cells incubated with epacadostat (1 µM) and monitored for 24, 48, and 72 hours by MTT assay. Vehicle-treated cells were used as control (ctrl). Cell viability is directly proportional to the absorbance measured at 570 nm. (C) Kynurenine (µM) released by SKOV-3 cells treated with epacadostat (1 µM) for 24 hours. Vehicle-treated cells were used as control (ctrl). (D) Immunoblot analysis of IDO1 protein expression in lysates from SKOV-3 cells treated as in (C). β-tubulin expression was used as normalizer. One representative immunoblot of three is shown. The IDO1/β-tubulin ratio of scanning densitometry analysis is reported as fold change of epacadostat-treated versus vehicle-treated cells (ctrl; dotted line, 1-fold). Data in (B-D) are mean ± SD of three independent experiments. Data in (B) were analyzed by two-way ANOVA followed by post-hoc Bonferroni’s test; data in (C, D) were analyzed by unpaired Student’s t-test. ns, not significant, **P < 0.01, ***P < 0.001. [file Image_1.jpeg]

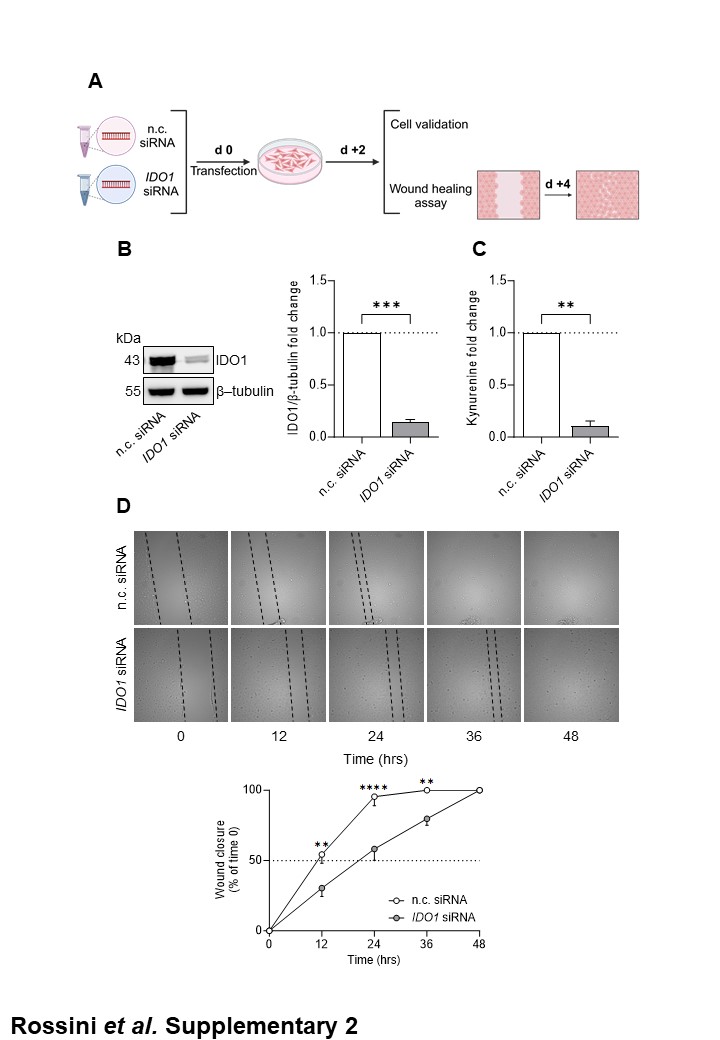

Supplement: Supplementary Figure 2 — IDO1 knockdown slows down SKOV-3 cells migratory capacity. (A) Experimental workflow for IDO1 knockdown, cell validation, and wound healing assay in SKOV-3 cells (Created by BioRender.com). SKOV-3 cells were transfected with IDO1-specific or negative control (n.c.) siRNAs (d 0; d, day) and after 48 hours (d +2) were either validated for IDO1 silencing, or used for wound healing assay and monitored for additional 48 hours (d +4). (B) Immunoblot analysis of IDO1 protein expression in lysates from SKOV-3 cells after 48 hours of siRNA transfection, as indicated in (A). β-tubulin expression was used as normalizer. One representative immunoblot of two is shown. The IDO1/β-tubulin ratio of scanning densitometry analysis is reported as fold change of IDO1 siRNA- versus n.c. siRNA-transfected cells (dotted line, 1-fold). (C) Kynurenine released by SKOV-3 cells treated as in (B). Results are shown as kynurenine fold change of IDO1 siRNA- versus n.c. siRNA-transfected cells (dotted line, 1-fold). (D) Analysis of the wound closure (black dotted lines) in SKOV-3 cells treated as indicated in (A), over the time (from 0 to 48 hours). For each reported time point (0, 12, 24, 36, 48 hours), one representative image of three is shown. Data are reported as percentage of the wound closure respect to time 0 (time 0 = 0%; dotted line, 50%). Data in (B-D) are mean ± SD of two independent experiments. Data in (B, C) were analyzed by unpaired Student’s t-test; data in (D) were analyzed using two-way ANOVA followed by post-hoc Bonferroni’s test. **P < 0.01, ***P < 0.001, ****P < 0.0001. [file Image_2.jpeg]
